# The importance of tacrolimus in the treatment of allergic keratoconjunctivitis

**DOI:** 10.1186/1939-4551-8-S1-A234

**Published:** 2015-04-08

**Authors:** Georgia Araujo, Mariana Nascimento, Patricia Arruda, Luiz Felipe Moraes, Marina De Andrada Carvalho, Décio Medeiros, Emanuel Sarinho

**Affiliations:** 1 Federal University of Pernambuco, Brazil

## Background

Keratoconjunctivitis (KC) is an eye disease that potentially leads to blindness if not treated properly, which often requires follow-up for more than an expert. The aim of this study is to evaluate the response of topical tacrolimus in allergic keratoconjunctivitis.

## Methods

A longitudinal study of series of cases conducted between September/2013 and June/2014, with 24 patients followed in Ocular Allergy ambulatory, Hospital das Clinicas of the Federal University of Pernambuco. The patients included in the study had symptoms such as: itching, burning, redness and/or edema uncontrolled medical monitoring initial and were then taken to the ophthalmologist for defining more specific treatment.

## Results

The median age was 10 years, with 15 male patients (62%). Of the 24 patients, 14 (58%) patients had allergic rhinitis, 7 (29%) had a diagnosis of asthma and rhinitis associated and 2 (8%) had rhinitis, asthma and atopic dermatitis. All were using medications for rhinitis and/or asthma continuously. Regarding the treatment of conjunctivitis, only 1 patient (4%) remitted symptoms using of mast cell membrane stabilizer, 6 (25%) have control of symptoms KC using mast cell membrane stabilizer and antihistamine, others 6 (25 %) was necessary to add an ocular lubricant. In 11 patients (46%) with severe allergic KC was necessary to associate a topic corticosteroids and 5 (45%) of these group became steroid-dependent, only reaching remission of symptoms with tacrolimus 0.03% ophthalmic ointment. All patients who used tacrolimus reported improvement in signs and symptoms and adverse events were limited to the local burning in one patient who interrupted treatment, despite that improvement of edema and hyperemia.

## Conclusions

Allergic rhinitis is very common in patients with KC, underscoring the importance of joint processing for clinical improvement. The use of topical tacrolimus 0.03% in and out of the conjunctival sac seems to be in the short term, effective, well tolerated and safe in the treatment of allergic conjunctivitis refractory to traditional treatment, and avoid prolonged corticosteroid therapy and its associated side effects, such as hypertension eyepiece.
